# Clinical neuromodulatory effects of deep brain stimulation in disorder of consciousness: A literature review

**DOI:** 10.1111/cns.14559

**Published:** 2023-12-19

**Authors:** Tianqing Cao, Shenghong He, Luchen Wang, Xiaoke Chai, Qiheng He, Dongsheng Liu, Dong Wang, Nan Wang, Jianghong He, Shouyang Wang, Yi Yang, Jizong Zhao, Huiling Tan

**Affiliations:** ^1^ Department of Neurosurgery, Beijing Tiantan Hospital Capital Medical University Beijing China; ^2^ China National Clinical Research Center for Neurological Diseases Beijing China; ^3^ Medical Research Council Brain Network Dynamics Unit, Nuffield Department of Clinical Neurosciences University of Oxford Oxford UK; ^4^ School of Information Science and Technology Fudan University Shanghai China; ^5^ Department of Neurosurgery Aviation General Hospital Beijing China; ^6^ Department of Neurosurgery Ganzhou People's Hospital Ganzhou Jiangxi Province China; ^7^ Chinese Institute for Brain Research Beijing China; ^8^ Beijing Institute of Brain Disorders Beijing China

**Keywords:** deep brain stimulation, disorder of consciousness, effectiveness, neuromodulation therapy

## Abstract

**Background:**

The management of patients with disorders of consciousness (DOC) presents substantial challenges in clinical practice. Deep brain stimulation (DBS) has emerged as a potential therapeutic approach, but the lack of standardized regulatory parameters for DBS in DOC hinders definitive conclusions.

**Objective:**

This comprehensive review aims to provide a detailed summary of the current issues concerning patient selection, target setting, and modulation parameters in clinical studies investigating the application of DBS for DOC patients.

**Methods:**

A meticulous systematic analysis of the literatures was conducted, encompassing articles published from 1968 to April 2023, retrieved from reputable databases (PubMed, Embase, Medline, and Web of Science).

**Results:**

The systematic analysis of 21 eligible articles, involving 146 patients with DOC resulting from acquired brain injury or other disorders, revealed significant insights. The most frequently targeted regions were the Centromedian‐parafascicular complex (CM‐pf) nuclei and central thalamus (CT), both recognized for their role in regulating consciousness. However, other targets have also been explored in different studies. The stimulation frequency was predominantly set at 25 or 100 Hz, with pulse width of 120 μs, and voltages ranged from 0 to 4 V. These parameters were customized based on individual patient responses and evaluations. The overall clinical efficacy rate in all included studies was 39.7%, indicating a positive effect of DBS in a subset of DOC patients. Nonetheless, the assessment methods, follow‐up durations, and outcome measures varied across studies, potentially contributing to the variability in reported efficacy rates.

**Conclusion:**

Despite the challenges arising from the lack of standardized parameters, DBS shows promising potential as a therapeutic option for patients with DOC. However, there still remains the need for standardized protocols and assessment methods, which are crucial to deepen the understanding and optimizing the therapeutic potential of DBS in this specific patient population.

## Graphical abstract

This graphical abstract provides an overview of the article, focusing on the clinical neuromodulatory effects of Deep Brain Stimulation (DBS) in Disorders of Consciousness (DOC) patients. The left schematic diagram illustrates the DBS implantation procedure within the patient's body, including the placement of the implantable pulse generator (IPG). On the right, details are presented regarding the utilization and distribution of different targets and frequencies. In summary, the comprehensive clinical response rate across all studies included in the analysis was 39.7%.

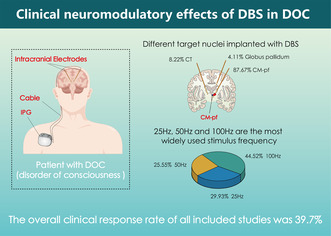

## 1 INTRODUCTION

Disorders of consciousness (DOC) present complex neuropsychiatric disturbances, arising from various causes such as trauma, stroke, or anoxia.^1^, ^2^ Within the spectrum of DOC, distinct conditions include coma, vegetative state/unresponsive wakefulness syndrome (VS/UWS), and minimally conscious state (MCS).^3^ Coma represents a profound absence of arousal and awareness, lacking any response to external stimuli. VS/UWS involves intermittent wakefulness without self‐awareness or environmental recognition, often accompanied by eye‐opening movements.^4^, ^5^, ^6^, ^7^ MCS patients exhibit consistent clinical movement, but their state of awareness is unstable, recently classified into MCS^−^ (displaying low‐level behavioral responses) and MCS^+^ (demonstrating high‐level language‐dependent responses)^2^, ^8^, ^9^ Understanding these distinctions is essential for accurate diagnosis and appropriate management of individuals with DOC.

Despite significant efforts in pharmacological treatment, invasive therapies, and physical rehabilitation,^10^ successful treatment of DOC remains limited due to a lack of comprehensive understanding of the underlying pathophysiology. An emerging avenue of exploration is deep brain stimulation (DBS), an invasive neurosurgical technique used for neuromodulation in patients with DOC. The origins of DBS for consciousness modulation trace back to pioneering experiments in 1949 by Moruzzi and Magoun, which revealed the correlation between thalamic or midbrain stimulation and the maintenance of brain arousal.^11^ In 1969, Hassler et al. explored chronic stimulation in patients with impaired consciousness. They observed some arousal effects and modest improvements during the 19‐day stimulation period, but there was no significant shift in the consciousness level.^12^

DBS parameters are adjustable and can be fine‐tuned to optimize the patient's responsiveness to self‐awareness and their environment, therefore, they have shown potential for improving motor function once consciousness level has improved. An intriguing case proposed by Schiff et al.^13^ suggested that DBS may facilitate functional recovery by compensating for impaired arousal regulation through central thalamus stimulation. While DBS holds promise as a therapeutic approach for DOC, its effectiveness and underlying mechanisms require further investigation. In addition, most individual studies have very small sample size, the overall effectiveness of DBS on DOC in all operated patients is still unknown.

Currently, the clinical application of DBS to treat patients with DOC poses several challenges, including patient selection, determination of the optimal timing for DBS treatment, and the selection of appropriate targets and stimulation parameters.^14^, ^15^, ^16^ A deeper understanding of the pathophysiological mechanisms underlying consciousness impairment will help address these issues. The lack of globally standardized clinical strategies also creates significant difficulties for clinicians and program managers in clinical settings. To better understand the pathophysiological mechanisms of DOC and to evaluate the overall effectiveness of DBS in treating DOC, we conducted a meticulous literature review, systematically summarizing the current state and challenges associated with DBS implementation for DOC.

## 2 METHODS

We conducted a systematic search of four electronic literature databases (PubMed, EMBASE, Medline, and Web of Science) using two key concepts: disorders of consciousness (DOC) and deep brain stimulation (DBS). The search terms included relevant keywords such as “DBS,” “deep brain stimulation,” “consciousness disturbance,” “disorders of consciousness,” “coma,” “vegetative state,” “unresponsive awakening syndrome,” and “coma insomnia.”

The inclusion criteria for this review encompassed English‐language literature reporting on the utilization of DBS in human patients as a treatment for DOC, including MCS, PVS, VS, coma, or other related persistent states impacting consciousness from 1968 to April 2023. Articles that did not discuss or report the use of DBS for treating DOC, but focused on other brain stimulation methods such as transcranial direct current stimulation (tDCS), repeated transcranial magnetic stimulation (rTMS), spinal cord electrical stimulation (SCS), vagal nerve stimulation, transcranial ultrasound stimulation (TUS), and transcranial laser therapy, were excluded. Studies that primarily employed DBS for treating other conditions such as dystonia, pain, tremor, or psychiatric symptoms following structural brain injury were also excluded. Initial screening of articles was based on their titles and abstracts, followed by a detailed examination of the full articles.^17^ Furthermore, the literature had to explicitly describe the surgical procedure and postoperative neuromodulation plan. We also conducted additional searches depend on the references of included articles to identify any other reports related to DBS treatment for DOC.^12^, ^18^

Published articles were summarized, and duplicates were removed. Ultimately, 21 studies were included and with a total of 146 patients. These articles, detailing DBS regulation strategies in clinical settings, were extracted and summarized for review (see Table 1).

**TABLE 1:** Overview of patient reports of DBS for DOC.

	References	Sample/Sex/Age (at injury)	Etiology	Diagnosis	Time from initial injury to DBS	Brain targets	DBS parameters			Mode of stimulation	Follow‐up after surgery (months)	Outcome
							DBS Vol.V	DBS Freq.Hz	DBS Pulse width			
1	McLardy et al. 1968	One male, 19	TBI	“Coma vigilans”	7	Left thalamus and left midbrain for intralaminar nuclei and reticular formation	na	250 Hz	1000	na	24	Could move left hand, No change in consciousness and Died 24 months after surgery
2	Hassler et al. 1969	One male, 26	TBI	“Apallic syndrome” or “coma vigil”	5 months	Basal portion of the left lateropolar nucleus of the thalamus Basal part of the right pallidum	Left: 20 V Right: 30 V	Left: 25–50 Right: 8	1000–3000	After 20 days the electrodes were removed Stim Int 0.25/6 or 0.25/8 (3–4 times/day)	<1	Improvement of consciousness Spontaneous movements of the left limbs Unintelligible vocalization
3	Sturm et al. 1979	One male, 68	Outcome of operation for aneurysm, probably ischemia of the brain stem	“Subcoma with unconsciousness”	<1 months	Right rostral part of the lamella medialis thalami Left nucleus reticulatus polars thalami	6–10 V	50	500	Bipolar After 4 days, the right side electrode was removed (without effect) Stim 10/12 DT (10:00 am to 8:00 pm) (10 min each hour)	2	Partial and temporary limited improvement After 2 months the patient died from pneumonia
4	Tsubokawa et al. 1990 (see also Katayama et al.)	Five male, 24, 43, 44, 48, 75 Three female, 41, 41, 74	4 TBI 3 vascular 1 anoxic	PVS (PCS 2–4)	>6* months	6 non‐specific thalamic nucleus (CMPf) 2 Nucleus cuneiformis in mesencephalic reticular formation Unilateral	0–10 V	50	na	Stim 2/22 DT (4 times 30 min)	12	3 “full recovery” (PCS 8–9) 1 incomplete recovery (PCS 7) 3 no recovery (PCS 3–5)
5	Hosobuchi & Yingling 1993	One male, 23	Anoxic (ischemia)	Apallic state, GCS <6	8 months	na	Na (amplitude increased till “30% of the arousal threshold”)	na	na	Stim 12/12 DT	10	Improvement Oral feeding possible Could respond to verbal commands Could not shake or nod the head on yes/no questions
6	Cohadon & Richer 1993	25 patients, (na)	na	VS	>3 months	CMPf	5–10 mV	50	500	2 monopolar and 23 bipolar Stim 12/12 DT (8:00 am to 8:00 pm)	12–144	1 improvement to moderate disability (GOS) 10 improvement to severe disability (GOS) 2 died 12 no recovery
7	Yamamoto et al. 2005	Four female, 19, 41, 58, 59 Three male, 30, 43, 75 One person, (na)	6 vascular 2 TBI	PVS (PCS 2–5)	3–6* months	19 CMPf 2 mesencephalic reticular formation Unilateral, less injured side	Various intensities	25	na	Bipolar Stim Int 0.5/3 DT	120	All 8 recovered: 7 are bedridden (PCS 8–10) 1 is “able to live in a wheelchair”
		Seven male, 29, 30, 42, 44, 48, 49, 56 Six female: 30, 39, 41, 44, 61, 74	7 TBI 3 vascular 3 anoxic									All 13 no recovery (PCS 3–7)
8	Schiff et al. 2007	One male, 38	TBI (closed head injury)	MCS	78 months	Bilateral, anterior intralaminar thalamic nuclei and adjacent paralaminar regions	4	(After titration final set of stimulation parameters) 100	na	Left monopolar, right bipolar Parameter optimization 70–250 Hz and 0–5 V 6 months double‐blind crossover study 3 times 30 subsequent days on, 30 days off If DBS is on, Stim 12/12 DT	24	CRS‐R subscales, various improvements (arousal, motor and communication as primary measures) Restoration of communication (“interact consistently and meaningfully”)
9	Yamamoto et al. 2002–2013	5 patients, 18–47 (mean = 33.5 ± 14.3)	3 TBI 2 vascular	MCS	3–6 months	5 CM‐Pf, unilateral, less injured side	Various intensities	25	na	Bipolar Stim Int 0.5/3 DT	120	All 5 recovered, live at home with family Severe disabled condition (GOS) Need wheelchair, 4/5 patients “could not operate wheelchair by themselves”
10	Wojtecki et al. 2014	One female, 38	TBI (closed head injury)	DoC (GCS 4)	84 months	Bilateral, CT (internal medullary lamina and the nuclei reticularis thalami)	4	70–250	na	Other parameters same as Schiff et al. 2007	na	No recovery (yet) Patient had increased brain activity on response to her children
11	Magrassi et al. 2016	One male, 58	TBI	MCS (CRS‐R 14)	28 months	Bilateral, anterior intralaminar thalamic nuclei and adjacent paralaminar regions	na	80–110 (median 100)	na	Bipolar Stim 14/10 DT (7:00 am to 9:00 pm)	18	CRS‐R 15
		One male, 23	TBI	VS/UWS (CRS‐R 8)	34 months						60	CRS‐R 11
		One male, 29	TBI	VS/UWS (CRS‐R 6)	96 months					Unipolar Stim 14/10 DT (7:00 am to 9:00 pm)	59	CRS‐R 9
12	Chudy et al. 2017	One; male; 17	Anoxic (CA)	MCS (C/NC 2.0/1)	2 months	Unilateral CM‐pf (preferentially on left side, but, if too damaged right side)	2.5–3.5	25	90	Monopolar Stim Int 0.5/2 DT	60	C/NC 0, aware Regained consciousness Largely independent
		One; male; 23	Anoxic (CA)	MCS (C/NC 1.8/1)	2 months						57	C/NC 0, aware Regained consciousness Largely independent, ‐“still experiences short‐term memory impairment and emotional regression”
		One; female; 15	TBI	MCS (C/NC 1.6/1)	11 months						51	C/NC 0, aware Regained consciousness “has a severe left side hemiparesis and needs assistance in everyday life” Needs wheelchair
		Seven male: 17, 17, 20, 25, 34, 43, 59 Four female: 16, 28, 39, 49	3 TBI 8 anoxic (CA)	1 MCS 10 VS	3–138 months						38–59	1 VS improved to MCS 3 died 7 no recovery
13	Lemaire et al. 2018	N1; male; 32	TBI	UWS (CRS‐R 6.1; after surgery before CT‐DBS ON)	146	Right pallidum + bilateral thalamus	6	30	60	Blind, 3‐month crossover (CO) phase, with two randomized periods of 1.5 months, 1.5‐month ON (CO‐ON) and OFF (CO‐OFF) Unblinded stimulation Phase of at least 5‐months (DBS‐ON) If DBS‐ON; Stim 24/24	8	CRS‐R 8.4 (41 average)
		N2; female; 62	Hemorrhagic strokes	MCS^−^ (CRS‐R 9.6; after surgery before CT‐DBS ON)	14	Bilateral pallidum + bilateral thalamus						CRS‐R 9.5 (34 average)
		N3; male; 24	TBI	MCS^−^ (CRS‐R 11.7; after surgery before CT‐DBS ON)	37							CRS‐R 13.8 (40 average)
		N4; female; 22	TBI	MCS^−^ (CRS‐R 4.8; after surgery before CT‐DBS ON)	48							CRS‐R 4.3 (28 average)
		N5; female; 47	hemorrhagic strokes	MCS^−^ (CRS‐R 4.2; after surgery before CT‐DBS ON)	27		4					CRS‐R 3.0 (27 average)
14	Gottshall et al. 2019	One; male; 17	TBI	MCS (CRS‐R 11.8; average; after surgery before CT‐DBS ON)	257	Bilateral CT (the sensory relay nucleus of the thalamus on both the right and left hemispheres)	4	100 (For the majority of the 7½ years); 130; 175 (6 month crossover phase)	90	Stim 12/12 DT (6:00 am to 6:00 pm) Left bipolar; right mono‐polar	96.5	MCS CRS‐R 11.8 (average) There was no change in CRS‐R scores between active CT‐DBS time points. Change in sleep pattern Recorded via EEG
15	Marina et al. 2021 (4 patients were the same as Chudy et al. 2017)	One; female; 18	TBI	UWS (C/NC, 2.6/2)	14	CM‐pf (preferentially on left side, but, if too damaged right side)	2.5–3.5	25	90	Monopolar Stim Int 0.5/2 DT	23	C/NC 0, aware Regained consciousness “has a severe left side hemiparesis and needs assistance in everyday life” Needs wheelchair
16	Hisse Arnts et al. 2022	One female, 38	TBI	MCS (CRS‐R 9–14)	96	Bilateral, CM‐pf	na (stimulation current was 2.5–3 mA)	50 (in second year); 130 (in first year)	450	Bipolar Stim Int 0.5/2 DT Stim in DT (when fre was 50 Hz)	24	CRS‐R 9–12 Increased arousal, visual pursuit, return of swallowing, and reduction of spasticity
17	Dang et al. 2022	5 male: 25, 52, 35, 49, 31 4 female: 35, 45, 11, 26	4 TBI 3 vascular 2 anoxic	MCS (CRS‐R 9–14)	6–12 months	Bilateral, CM‐pf	3	100	120	Bipolar Stim Int 15/20 (min) DT (from 8:00 a.m. to 8:00 p.m.) A randomized sham‐controlled crossover study. In the first group. Five patients received 100 Hz DBS and sham DBS on two separate days separated by 24 h. The same treatment was given to the second group but in reversed order (no. of patients = 4)	6	The CRS‐R scores of P4 from 9 to 12, 8 to 11 for P7, and 9 to 16 for P9 EEGs show that the brain functional connectivity of P4, P7 and P9 significantly improved after DBS
18	yang et al. 2023	23 male; 14 female	8 trauma; 10 Anoxia; 19 Stroke	13 MCS; 24 VS/UWS	3–5 months 25 (67.6%); 6–11 months 8 (21.6%); ≥12 months 4 (10.8)	Bilateral, CM‐pf	1.0–4.0	100	120	The IPGs were activated 7 days after surgery Stimulation was provided continuously from 08:00 to 20:00 h with a 15 min on/off cycle (15 min on, 15 min off)	12	12 patients (10 in MCS; 2 in VS/UWS) improve in consciousness at 1 year (more than 3 points)
19	shu et al. 2023	7 male: 48, 37, 60, 46, 61, 53, 55; 3 female: 72, 75, 69	4 trauma; 4 hemorrhage; 2 anoxic	2 MCS; 8 VS	3–18 months	4 patients right CM‐pf nuclei; 6 patients bilateral CM‐pf nuclei	2.5–3.5	100	120	15‐min period every 30‐min during the daytime	1	Six patients showed increased CRS‐R scores; P3, P4, and P6 changed from VS to MCS^−^

Abbreviations: CA, cardiac arrest; CM‐pf, Centre Median Parafascicular complex; CRS‐R, JFK Coma Recovery Scale Revised; CT, “central thalamus”; DBS, deep brain stimulation; DOC, disorders of consciousness; DT, stimulation during Day Time; GCS, Glasgow Coma Scale; GOS, Glasgow Outcome Scale; MCS, Minimally Conscious State; na, not available; PCS, Prolonged Coma Scale; PVS, Persistent Vegetative State; Stim Int, Intermittent Stimulation in hours ON/total period in hours; Stim, stimulation cycle of DBS in hours ON/ hours OFF; TBI, traumatic brain injury; UWS, Unresponsive Wakefulness Syndrome; VS, vegetative state.

## 3 RESULTS

### 3.1 General description

#### 3.1.1 Patient selection

**FIGURE 1:**
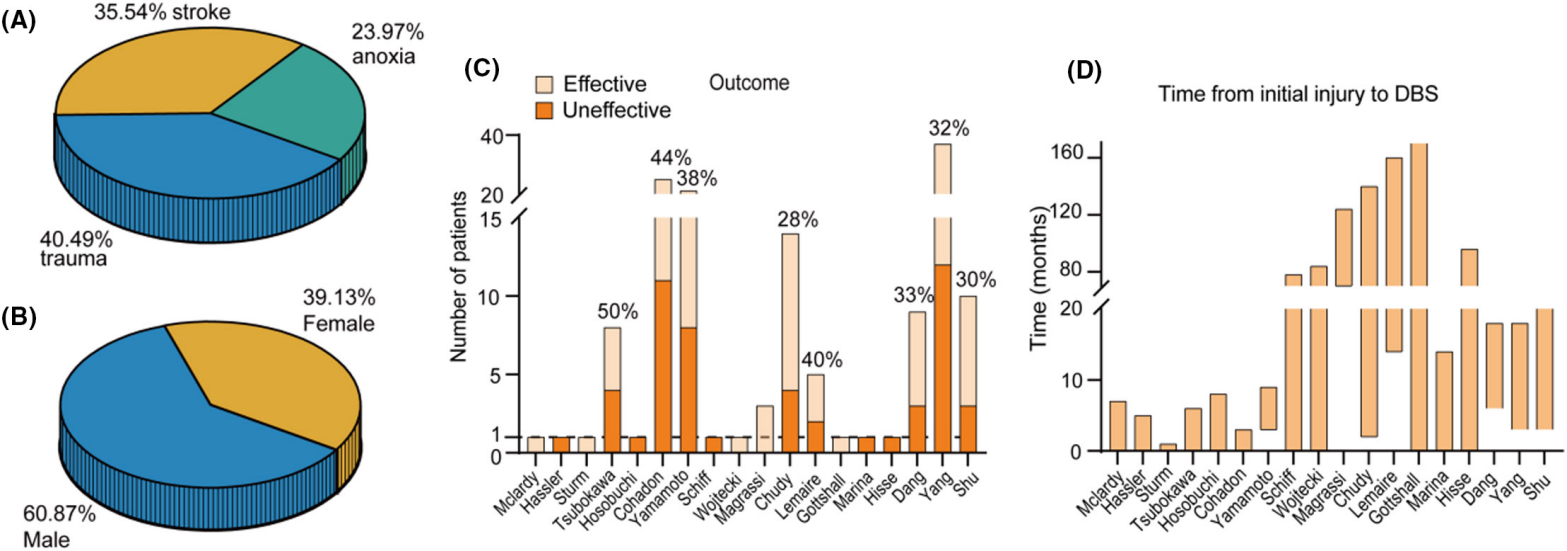
Summary of the clinical characteristics of patients with Disorders of Consciousness (DOC). (A) The proportions of different etiologies included in all studies. (B) The gender ratio of these patients. (C) The efficacy rates of Deep Brain Stimulation (DBS) reported in individual studies. (D) The time interval between brain injury and surgical treatment across all studies. The *X*‐axis represents the literature sorted from left to right according to publication date. The orange bar in D indicates the time window for surgical procedures.

After extracting the data from the literature, except for Cohadon's study which did not provide information on the etiology of the patients (*n* = 25), a total of 121 patients with DOC underwent DBS surgeries in 11 clinical centers in 8 countries. The causes of DOC in the operated patients include trauma (40.5%, *n* = 49), stroke (35.5%, *n* = 43), and anoxia (24%, *n* = 29) (Figure 1A). Male patients constituted 60.9% (*n* = 70), while female patients accounted for 39.1% (*n* = 40) (Figure 1B) of the total sample. All reported patients aged between 15 and 75 years old, with a mean age of 40.71 ± 16.94 years. Among them, 33.3% (*n* = 27) were between 15 and 30 years old, 53.1% (*n* = 43) were between 31 and 60 years old, and 13.6% (*n* = 11) were over the age of 60. Diagnosis of vegetative state/unresponsive wakefulness syndrome (VS/UWS) accounted for 50.6% (*n* = 44) of the cases, while minimally conscious state (MCS) diagnosis accounted for 49.4% (*n* = 43) (considering data after the introduction of the concept of MCS in 2002).

#### 3.1.2 Overall effectiveness of DBS treatment

The overall effectiveness of DBS for patients with DOC was 39.7% (58/146), as measured by improvements in clinical behavior scale scores in different studies, such as improvement in movements, responsibility to verbal commands, increased coma recovery scale‐revised [CRS‐R] scores, Glasgow coma scale [GCS] scores,^19^ Glasgow outcome scale score [GOS] scores, or standard Rappaport Disability Rating [RDR] scale, and Coma/Near Coma [C/NC] scale (Figure 1C).^20^, ^21^ Among all studies, Tsubokawa et al. reported the highest effectiveness at 50% (4/8),^22^ while Chudy et al. had the lowest effectiveness at 28.6% (4/14),^20^ excluding case studies in McLardy, Hassler, Sturm, Hosobuchi, Schiff, Wojtecki, Gottshall, Marina Raguz, and Hisse Arnts (details in Table 1).

#### 3.1.3 Preoperative evaluation

All the studies had specific criteria for patient inclusion and exclusion. These criteria varied across studies but generally involved clinical scale assessments (CRS‐R, GCS, GOS, RDR, or C/NC) to classify patients' state of consciousness into VS/UWS or MCS. Neuroimaging techniques (CT, MRI, PET, fMRI, MEG) were used, as well as neurophysiological assessments (EEG, somatosensory evoked potentials [SEP], motor evoked potentials [MEP], mismatched negativity [MMN], brainstem auditory evoked potentials [BAEPs], functional near‐infrared spectroscopy [fNIRS]).^20^, ^21^, ^22^, ^23^, ^24^, ^25^, ^26^, ^27^, ^28^, ^29^, ^30^ Strict exclusion criteria were applied, including infectious or metabolic brain injury, additional neurological diseases not related to DOC, life expectancy of less than 1 year due to non‐neurological disease, persistent incurable infections, pregnancy, use of sedative drugs, thalamus site injuries indicated by imaging data, and legal representative requests to exclude patients from the study.^23^, ^28^, ^31^

#### 3.1.4 Time window for surgical procedures

Patients undergoing DBS surgery ranged from 1 month to over 20 years after being diagnosed with DOC (Figure 1D). To ensure the stimulating effect was not affected by self‐recovery, patients included in the study underwent DBS treatment at least 3–6 months after brain injury.^28^, ^29^, ^31^ Except for Sturm's study, the operation took less than 1 month. Some patients with MCS who received DBS treatment 21 years after injury can still observe late and progressive alterations of sleep dynamics.^27^

#### 3.1.5 Stimulation target

**FIGURE 2:**
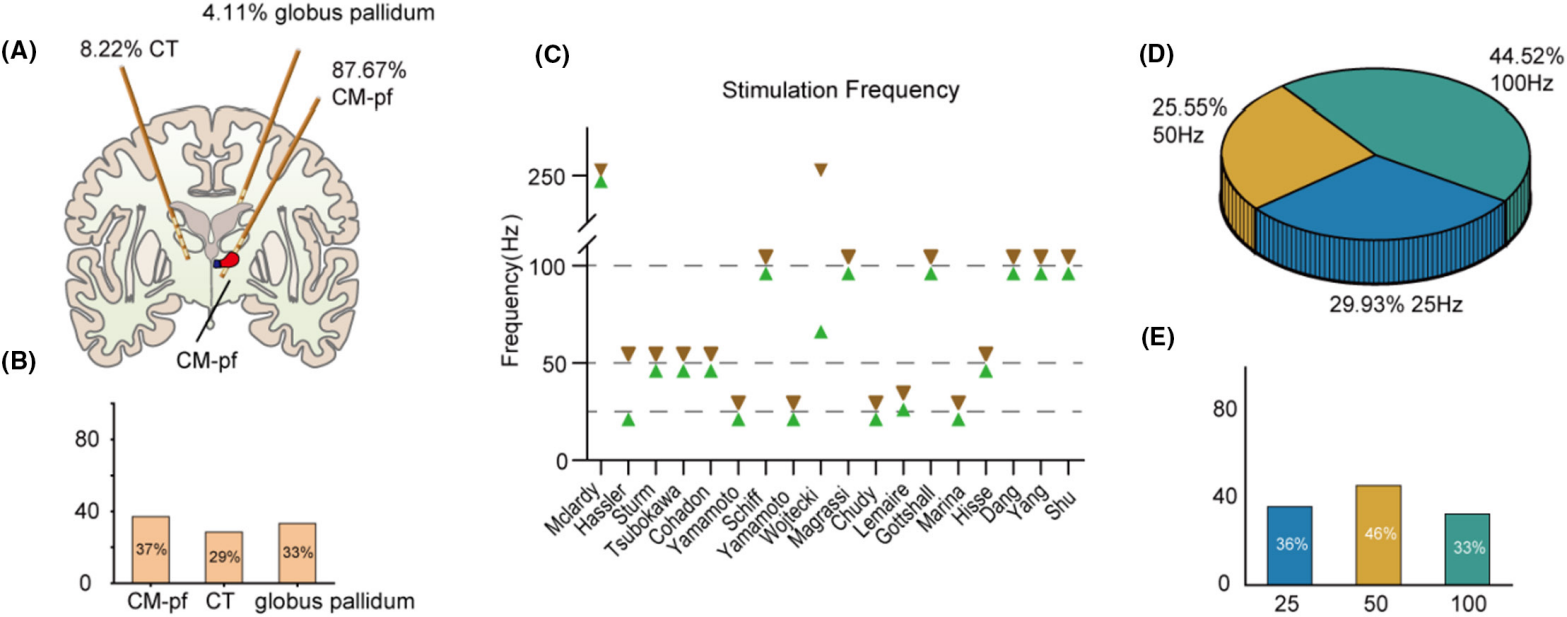
Stimulation targets and stimulation frequency selected in all the researches. (A) The proportions of different target nuclei implanted with DBS. (B) Efficacy rates after stimulation of different target nuclei. (C) Variations in stimulation frequencies across different studies. (D) Proportions for frequencies set at 25, 50, and 100 Hz respectively. (E) Averaged efficacy rates for different stimulation frequencies.

The target brain areas for DBS varied among the selected studies. The most common target was Centromedian‐parafascicular complex (CM‐pf), with a total of 128 patients (87.67%) in all reviewed studies. There were also 12 patients (8.22%) treated with central thalamus (CT) DBS, and 6 patients (4.11%) with DBS targeting globus pallidum (Figure 2A,B). Among those receiving CM‐pf stimulation, 101 patients underwent bilateral implantation, while 26 patients received unilateral implantation, with 20 preferentially left‐sided and 4 preferentially right‐sided, and 2 patients in Cohadon's study did not provide the left or right side of the implantation. Unilateral hemisphere implantation, particularly on the left side, has been the prevailing choice among most studies. However, there is a recent trend toward bilateral hemispheres implantation to enable simultaneous stimulation on both sides of the brain. In another study, researchers targeted pallidum and thalamus for left and right hemispheres (*n* = 5 patients) with one pulse generator on the chest wall and another on the abdominal wall.^32^ For central thalamic and globus pallidum stimulation, bilateral implants were the preferred approach. Based on these studies, DBS targeting CM‐pf, CT, and globus pallidum had averaged efficiencies of 37.09%, 28.57%, and 33.33%.

### 3.2 Selection of stimulation parameters

#### 3.2.1 Post‐operative stimulation timing

In all studies, patients were allowed time to recover from the DBS surgeries, with post‐operative stimulation timing ranging from 3 days to 2 months. To avoid brain tissue edema around the electrodes and the impact of bleeding on impedance, most studies started the stimulation 7–14 days after surgeries. However, some studies (*n* = 10 patients) applied constant monopolar stimulation from as early as the third day after DBS implantation.^30^ Lemaire et al conducted a DBS titration phase for a month, while Schiff et al exposed the patient to different stimulation patterns for 2 months in order to identify the optimal behavioral responses.^13^, ^32^

#### 3.2.2 Stimulation contacts setting

The standard configuration for DBS electrodes involves quadripolar electrodes with four contacts at the probe's tip. This arrangement allows for precise shaping of the electric field along the *z*‐axis by programming different combinations of anodes and cathodes. The selection of stimulation contacts is mainly based on the experience of the clinicians and observation of stimulation responses, with necessary adjustments made according to clinical outcomes.^22^, ^33^ In some cases, patients have chosen specific configurations for their bilateral electrodes, such as selecting the lowest contact point on the left electrode as the cathode and using the highest contact point as the anode, with similar choices for the right electrode.^23^

While some evidence suggests that bilateral stimulation may not significantly enhance consciousness improvement for patients, further detailed comparative studies are needed to clarify this aspect. The introduction of directional electrodes since 2015 has allowed for more versatile shaping of the electric field, leading to an improved therapeutic window by enhancing efficacy and reducing adverse effects.^34^ This advancement has the potential to optimize the outcomes of DBS treatments for patients with disorders of consciousness.

#### 3.2.3 Frequency setting

DBS stimulation frequencies have undergone extensive investigation, with a wide range of frequencies reported across various studies, spanning from 8 to 250 Hz (Figure 2C). However, in clinical practices, the most commonly used stimulation frequencies are 25 Hz (29.93%, *n* = 41), 50 Hz (25.55%, *n* = 35), and 100 Hz (44.52%, *n* = 61) (Figure 2D). The averaged effective rates for these frequencies were 36.11% (25 Hz), 45.71% (50 Hz), and 32.76% (100 Hz) (Figure 2E). But since 2005, the 50 Hz has rarely been used, except for the Hisse Arnts's case study. However, Hisse Arnt's case study demonstrated that MEG following low‐frequency stimulation is significantly more similar to that of healthy control subjects compared to high‐frequency deep brain stimulation.^31^ When only considering studies focused on the CM‐pf since 2005, which involved 93 patients from 7 studies, the reported effectiveness was 36.1% (13 out of 36 patients) for 25 Hz stimulation and 31.6% (18 out of 57 patients) for 100 Hz stimulation, suggesting both 25 and 100 Hz frequencies show promising effects in improving consciousness in patients with DOC.

#### 3.2.4 Voltage and pulse width

**FIGURE 3:**
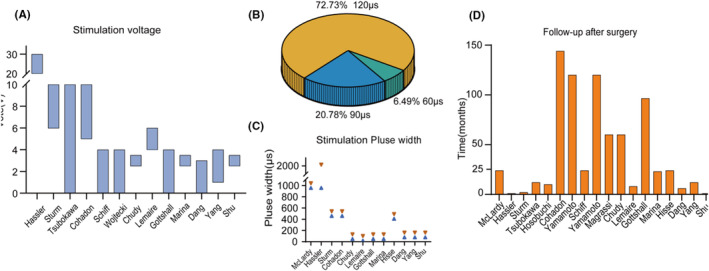
Stimulation vote, stimulation pulse width selected in all the researches and the follow‐up after surgery. (A) Variations in stimulation voltage across different studies, each bar indicates the voltage range that was applied within each study. (B) Proportions for pulse widths set at 60, 90, or 120 μs from the research of Chudy in 2017. (C) Differences in pulse width settings among various research (Only articles referring to pulse width are shown here). (D) Follow‐up durations after surgery for all studies. The *X*‐axis represents the literature sorted from left to right according to publication date.

In most studies, DBS utilizes a constant stimulation voltage, typically ranging from 0 to 30 V. However, there is a lack of standardization in voltage selection, and it is usually determined based on titration parameters and the patient's individual response to stimulation. Recently, researchers have adopted a more consistent approach by stabilizing the stimulus voltage at 3–4 V for improved reliability (Figure 3A). Additionally, in some cases, constant current stimulation has been employed. For example, Hisse Arnts et al.^31^ applied a constant current ranging from 2.5 to 3.0 mA to stimulate the CM‐pf and reported a more favorable recovery of consciousness.

Regarding pulse width, many studies lack specific descriptions of the pulse width used for stimulation. In earlier studies, a very high pulse width was commonly utilized. However, more recent studies mainly used a pulse width of 60 μs (6.49%, *n* = 5), 90 μs (20.78%, *n* = 16), or 120 μs (72.73%, *n* = 56) (Figure 3B,C). Notably, in a recent study of DBS targeting CM‐pf with a 1‐year follow‐up, the stimulation pulse width was adjusted to 450 μs, with no significant adverse effects compared to lower pulse widths.^31^ These results highlight the need for further research to determine the optimal pulse width for DBS treatment in patients with DOC. Standardization of pulse width could contribute to improved therapeutic outcomes and patient safety during DBS interventions.

#### 3.2.5 Stimulation period

In most studies, the focus has been on aligning DBS stimulation with circadian rhythms, leading to limited stimulation during the day. The most commonly used stimulation periods involve alternating periods of stimulation and rest. Specifically, two common patterns are employed: 15 min of stimulation followed by 15 min of rest (*n* = 56 in 3 researches), and 30 min of stimulation followed by 90 or 120 min of rest (*n* = 50 in 6 researches). These cyclic patterns are designed to optimize the effects of DBS by minimizing potential side effects and maximizing the therapeutic benefits during specific periods of the day.

### 3.3 Postoperative follow‐up

In all reviewed studies, postoperative follow‐up periods varied widely, ranging from 19 days to over 10 years, with a median follow‐up of 2 years (Figure 3D). The immediate postoperative follow‐up primarily focused on assessing the response to stimulation, which often resulted in increased patient awareness levels, improved motor control, and behavioral enhancements akin to acute arousal effects.

Long‐term follow‐up evaluations predominantly involved changes in clinical scales such as the Coma Recovery Scale‐Revised (CRS‐R) score or the Rappaport Disability Rating (RDR) scale, the Coma/Near Coma (C/NC) scale, and neuroimaging and neuroelectrophysiological assessments. Various studies used specific criteria to identify effective stimulation responses. For example, the Tsubokawa study considered increased coma scale scores and the ability to communicate through language or body motor as indicators of effective stimulation.^22^ Schiff et al. defined effective stimulation as an increase in CRS‐R score, improved motor abilities, and presence of communication.^13^ Yang's research considered a CRS‐R score increase of more than 3 points or a change in diagnosis from vegetative state (VS) to minimally conscious state (MCS) as indicative of effective stimulation.^29^

In addition to clinical measures, various neuroimaging techniques were employed to assess changes in brain activity and connectivity following DBS treatment. These included quantitative MRI analysis, functional MRI (fMRI), and functional near‐infrared spectroscopy (fNIRS), which reflected alterations in blood oxygen levels and metabolic activity in different brain regions. Positron emission tomography‐computed tomography (PET‐CT) was used to evaluate changes in neuronal metabolic activity across brain areas. Sleep pattern monitoring using electroencephalogram (EEG) allowed tracking of changes in sleep patterns, while functional brain connectivity shown in EEG indicated alterations in connections between different brain regions.

A retrospective analysis of postoperative follow‐up data was used to propose a prediction model for DBS treatment efficacy, adding to the comprehensive evaluation of DBS outcomes.^29^ These diverse evaluation metrics contribute to a more comprehensive understanding of the long‐term effects and potential benefits of DBS for patients with disorders of consciousness.

## 4 DISCUSSION

The medical community is increasingly directing its efforts toward DOC and collaborating to advance neuroimaging and neuro‐modulation technologies. This concerted approach has led to notable advancements in the diagnosis, prognosis prediction, and treatment of DOC.^4^, ^5^, ^35^ DBS known for its effectiveness in treating conditions like Parkinson's disease, primary tremor, and motor disorders,^14^ has shown promise in treating DOC as well, though the exact underlying mechanism remains unclear.^16^, ^36^ While significant progress has been made in understanding and managing DOC, there are still challenges to overcome, particularly in standardizing DBS parameters and treatment protocols. The heterogeneity in patient populations and differences in DBS targets and stimulation parameters hinder the establishment of consensus in clinical practice. However, researchers are striving to identify patterns and trends that can improve our understanding of effective neuromodulation and optimize the therapeutic potential of DBS for patients with DOC.

### 4.1 Patient selection

The cause for DOC and the conscious/awareness status may be important for patient section for DBS as a potential treatment. Most literature on DBS for DOC lacks a clear description of outcomes and prognoses for different causes of DOC, such as trauma, stroke, and anoxia. This knowledge gap makes it challenging to determine which type of DOC patients would benefit more from DBS treatment. In terms of conscious status, studies by Yamamoto and Yang have highlighted that patient diagnosed with minimally conscious state (MCS) before DBS surgery experienced greater improvements in CRS‐R scores compared to those diagnosed with vegetative state (VS).^25^, ^29^, ^37^

The CRS‐R scale plays a crucial role in diagnosing and grading DOC, distinguishing between VS and MCS. However, because the course of DOC can evolve over time, up to 40% of MCS patients without communication may be misclassified as VS.^4^ Regular evaluation of clinical behavior and dynamic patient classification are recommended to ensure accurate diagnosis and treatment adjustments. Routine imaging evaluations, such as CT or MRI, can help to keep checking the location of the patient's brain injury and assess the integrity of the thalamic nucleus, and to improve the diagnosis.

In the past two decades, several neuroimagings have been developed to detect awareness in patients with DOC using PET, fMRI, and EEG. Objective documentation of CNS damage after acquired brain injury is made possible by neuroimaging.^38^ Structural imaging MRI is the preferred method for visualizing the location and extent of brain damage in chronic DOC.^39^ Activation fMRI studies, utilizing auditory, tactile, or visual stimuli, have demonstrated near‐normal high‐level cortical activation in MCS and low‐level activation in VS.^40^ Recently developed quantitative diffusion tensor imaging (DTI) techniques, which permit the assessment of structural white matter damage, have emerged. Massive decreases in brain metabolism were demonstrated in DOC during ‘resting state’ conditions of ^18^F‐fluorodeoxyglucose PET (FDG‐PET) studies. In VS, FDG‐PET typically exhibits a decrease in brain function to 40%–50% of normal levels.^41^ In the context of DOC, EEG can be utilized to predict outcomes, assess residual cognitive function, discern consciousness, and offer a method to communicate with the external world without employing muscular channels.^42^ Transcranial magnetic stimulation (TMS) of the motor cortex, coupled with EMG response detection (motor ERPs), is utilized to evaluate cortical excitability, which is diminished in DOC and correlates with the level of consciousness.^43^, ^44^ However, they are not yet “recommended” in clinical guidelines due to their limited specificity and sensitivity.^45^, ^46^

As research in the field progresses, efforts to enhance diagnostic accuracy and refine treatment approaches for different etiologies of DOC are ongoing. By addressing these challenges and incorporating advanced neuroimaging techniques, clinicians may achieve more personalized and effective DBS interventions for patients with disorders of consciousness.

### 4.2 Time window for surgical procedures

When should we start considering DBS as an intervention for our patients? The PVS Multiple Association Working Group reviewed a series of VS patients with traumatic brain injury (TBI) and non‐TBI, revealed that the possirobability of TBI recovering consciousness from VS after 3 months was 33%, rising to 46% at 6 months, and further increasing to 52% after 12 months. In contrast, the likelihood of spontaneous recovery of consciousness for non‐traumatic VS patients was particularly low at only 11% after 3 months and rising to 15% after 12 months.^47^ Therefore, clinicians need to consider when a patient is most likely to spontaneously regain consciousness before starting DBS treatment, as this can interfere with our ability to accurately distinguish between spontaneous recovery and neural regulation. This requires individualized assessment while considering potential outcomes of active intervention through surgery, and reduction of long‐term complications.

Patients in all studies experienced significant impairment of consciousness that lasted from less than 1 year to more than 20 years. This is an initial challenge for DBS as a treatment for DOC, given current limitations in technology development and clinical evaluation. To address this issue, clinical guidelines have been developed to screen potential patients.^45^, ^46^

### 4.3 Target selection

Regardless of its cause, DOC is characterized by a widespread cessation of excitatory synaptic activity throughout the entire cerebral cortex.^2^ Recovery from coma relies on cellular and circuit mechanisms that restore excitatory neurotransmission through connections between the cortex, thalamocortical system, and thalamus.^1^, ^48^, ^49^, ^50^, ^51^ In cases of structural brain injuries, the mescocircurt mode proposes that recovery occurs through central neurons in the thalamus and their connections to frontal‐striatal circuits. Deep brain stimulation (DBS) can be used as a surgical tool to directly measure pathological brain activity and provide adjustable stimulation to restore dysfunctional circuits while compensating for lost arousal regulation typically controlled by intact frontal lobes.^2^, ^34^, ^49^ Current targets for clinical stimulation include:

#### 4.3.1 CM‐pf

CM‐pf nucleus refers to Centromedian‐parafascicular complex located within the internal medullary lamina (IML), an essential part within the thalamic intralaminar nuclei group situated between medial and lateral nuclear groups in the thalamus.^52^, ^53^, ^54^ CM‐pf is commonly referred to as the “loop nucleus” of the basal ganglia, which serves as an excitatory input for this system. Within the “mescocircurt”, the basal ganglia consist of loops formed by projections from the cortex and thalamus to the input stage of the basal ganglia, the striatum.^33^, ^55^, ^56^ In the thalamus, a large number of neurons in the neighboring CM and PF are directed to the striatum within the thalamus. These signals are then sent through cascade inhibitory nuclei to cortical projections in thalamic nuclei.^57^, ^58^ By stimulating the CM‐pf nucleus, the inhibitory effect of the basal ganglia on the thalamus is alleviated, thereby restarting the integration function of the thalamus in the forebrain‐cortical‐thalamic system. The results of this review showed that CM‐pf stimulation had best effective ratio for DOC. Adjusting the stimulation parameters after surgery resulted in effective clinical behavioral improvements, as well as improvements in neuroimaging and electrophysiology. (*n* = 127, efficiency is 37.1%).

#### 4.3.2 CT‐central thalamus region

The anatomical region containing the CM‐pf nucleus is also a DBS target of interest known as the “central thalamus” (CT) (*n* = 17, efficiency was 28.57%). The CT is connected to both the brainstem and frontal cortex, making it an important hub for both the “arousal system” and “executive function”.^38^, ^49^, ^54^ Researchers believe that targeting the CT is more suitable than targeting other thalamic targets such as CM‐pf because it can better support integrated remote network interaction^1^, ^55^, ^59^, ^60^ Schiff et al observed significant improvements in behavior after stimulating the central thalamus, including increased arousal levels, improved motor control, and enhanced behavioral persistence.^13^ These improvements may be due to direct activation of the frontal cortex and basal ganglia systems by neurons in the anterior medial and adjacent parametric regions of the confluent thalamus. These neurons act as key intermediate systems and common final pathways in both the brainstem arousal system and frontal regions, enabling executive control of effort regulation, working memory, selective attention, and vitality.^61^

#### 4.3.3 Other targets

In addition to the CM‐pf nuclei group and CT, there have been individual studies on stimulation of the pallidum and lateral thalamus with very strong arousal responses observed but no improvement in consciousness during 19 days of continuous stimulation.^12^ Chronic stimulation of the left nucleus reticulatus polaris thalami has also been shown to be effective; Not only was the autonomic reactions and behavioral arousal reactions observed after stimulation, but the long‐term results showed an increase in the patient's awareness level for a period of 7 weeks. After 2 months of stimulation, the autopsy conducted after the patient's death found that compared to corresponding areas on the right‐side contact part displayed extra‐insular cut loss outside ventricular membrane contour, moderate increases were also found in subventricular astrocytes which proved that the thalamic reticular nucleus played a role in enhancing levels of consciousness through non‐specific activation.^18^

#### 4.3.4 Considerations for target selection

The choice of DBS target for patients with DOC depends on various factors, including the underlying cause of the disorder, individual patient characteristics, and neuroimaging findings. The two main targets, the Centromedian‐parafascicular complex (CM‐pf) and the Central Thalamus (CT), have shown promising results in improving arousal levels and behavioral responsiveness in patients with DOC.^13^, ^22^, ^28^, ^29^, ^31^, ^45^ CM‐pf stimulation can directly influence the thalamocortical system, while CT stimulation has been associated with improved executive function and arousal regulation.

It is important to note that the optimal target may vary from patient to patient, and in some cases, stimulation of multiple targets may be considered for better outcomes.^29^, ^32^ Moreover, the development of directional electrodes has provided new possibilities for fine‐tuning the electric field and achieving more precise and selective stimulation of specific brain regions, potentially enhancing the therapeutic effects of DBS.^34^

Future research should aim to elucidate the underlying mechanisms and functional connectivity associated with each target. Comparative studies investigating the efficacy of different targets in specific subgroups of patients based on etiology, duration of DOC, and severity will further refine the selection process and optimize treatment outcomes. Additionally, advances in neuroimaging techniques, such as PET, fMRI, and EEG, could contribute to the identification of patient‐specific biomarkers that may aid in target selection and treatment planning.^2^, ^4^, ^5^, ^8^, ^9^, ^28^, ^62^, ^63^

### 4.4 Stimuli parameter selection

Selecting the appropriate patient and implanting the hardware are only the first steps toward a successful DBS therapy. The principal goal of programming is to maximize the effect of DBS on the fibers that underlie the beneficial effect of the therapy and avoid the recruitment of fibers related to adverse effects at the lowest possible energy costs.^14^, ^36^ Choosing different electrode contacts is a remedy for suboptimal electrode placement during surgery. When treating a target area that is close to an area that may cause adverse effects, it is necessary to adjust the electric field or guide the current in the desired direction for optimal stimulation.^34^ In monopolar stimulation, the active contact is set as negative or cathode, while the Implantable Pulse Generator (IPG) is set as positive or anode. This creates a broad electric field with a relatively equal distribution of excitations in all directions. However, in bipolar stimulation, another electrode contact point serves as an anode which minimizes current spread and produces a narrower area of stimulation.^36^

The stimulus frequency is an important parameter in the regulation process. It can be classified as high‐frequency or low‐frequency. Elevated frequency typically refers to above 100 Hz, while low frequency corresponds between 20 and 50 Hz.^21^, ^32^, ^64^, ^65^ High‐frequency stimulation studies have shown that muscle tone changes occur in DOC patients with partial relief and reduction or disappearance of muscle spasms, suggesting a beneficial effect on spasticity. Electrophysiological results showed widespread EEG desynchronization effects.^23^ In patients with low‐frequency stimulation, electrically stimulating the CM‐pf complex induces incremental recruitment and enhanced response; For extended periods of time after injury, low‐frequency DBS leads to clear and reproducible clinical changes. Low‐frequency stimulation can activate larger volume regions (VTA), enhancing brain functional connectivity and neural plasticity resulting in improved awakening as well as improvements in visual pursuit, spasms, and swallowing despite no improvement in behavioral performance.^24^, ^31^ It is suggested that DBS reactivates some of the disrupted neural networks, but the damaged brain still lacks the ability to adapt to changing cognitive demands.^1^, ^21^ Animal studies on DBS have yielded similar results, indicating that smaller thalamic stimulation (higher nuclear density) may be more susceptible to non‐specific diffusion of DBS currents around the intended target area.^1^

Typically, the impedance around the electrode stabilizes a few weeks after implantation. Any shift in the impedance results in a shift in the current flow. Recent studies have typically used a stimulation voltage range of 0–4 V to minimize adverse reactions in patients.^14^, ^34^ However, it is unlikely that a similar clinical response can be achieved across different patients, even with the same electrode settings and stimulation voltages. Constant‐current pulse generators automatically regulate the voltage to accommodate current changes caused by varying impedances and exhibit significant awakening effects, although there is no enhancement in CRS‐R scores.^31^ The relationship between amplitude and pulse width is nonlinear. While pulse width parameters include 60, 90, and 120 μs, presently 120 μs is most commonly utilized from the research of Chudy in 2017.^15^ There is a close relationship between sleep and consciousness due to shared anatomical structures and functional networks. Studies focusing on patient sleep dynamics have revealed that maintaining an approximately physiological sleep‐wake cycle and consistent sleep patterns leads to better outcomes in subacute cases of conscious dysfunction and higher residual function in chronic cases. Preliminary studies suggest that restoring normal sleep characteristics and promoting a regular sleep‐wake cycle may improve awake behavior.^27^, ^66^

### 4.5 Postoperative Follow‐up

Postoperative follow‐up plays a crucial role in assessing the effectiveness and long‐term outcomes of DBS therapy in patients with DOC. Various studies have reported that DBS induces an arousal effect at the beginning of stimulation, indicating accurate targeting of the brain region but without directly leading to consciousness recovery.^24^, ^26^ Therefore, it is essential to differentiate between the initial arousal response and sustained improvements in consciousness levels.

To ensure rigorous and unbiased clinical conclusions, some studies have employed double‐blind trial protocols, extensive baseline assessments, and carry‐forward effect detection.^13^ These measures help in evaluating the true impact of DBS therapy without confounding factors. However, challenges related to carry‐over effects have been observed, which may influence results during crossover phases.^48^ Some patients who had their DBS device removed at long‐term follow‐up still experienced excellent outcomes, suggesting that temporary stimulation reshapes neural pathways and restores connections between the thalamus and cortex. For others who relying on continuous DBS, stopping the stimulation led to a significant decrease in consciousness level, highlighting the importance of electrical stimulation in maintaining impaired brain networks.^66^

Long‐term follow‐up studies have demonstrated steady recovery of consciousness after DBS, typically occurring 3–6 months after stimulation and beyond. Neuroelectrophysiological assessments have indicated improvements in EEG functional connectivity and network parameters after 6 months of DBS, leading to enhanced consciousness levels, reshaped sleep characteristics, and improved behavioral responses.^28^ Neuroimaging findings have revealed increased regional cerebral blood flow (r‐CBF) and regional cerebral metabolic rate of oxygen consumption (r‐CMRO2) during stimulation, along with increased neurotransmitters and their metabolites in the cerebrospinal fluid of effective cases.^22^ Moreover, structural volume analysis has shown significant volume increases in specific brain regions, suggesting that chronic DBS impacts brain substance metabolism, functional connectivity, and induces neuroplasticity in the central nervous system.^21^

Among the patients studied, those with Minimally Conscious State (MCS) tend to have better outcomes compared to those with Vegetative State/Unresponsive Wakefulness State (VS/UWS). MCS patients often retain overall connectivity and essential brain networks, providing a foundation for subsequent functional recovery through DBS application.^29^

In conclusion, postoperative follow‐up studies have provided valuable insights into the long‐term effects of DBS therapy in patients with DOC. While initial arousal responses are observed, sustained improvements in consciousness levels are achieved over time through the reshaping of neural pathways, improved functional connectivity, and neuroplasticity in the brain. Understanding the mechanisms underlying these effects will further enhance the therapeutic potential of DBS in improving the quality of life for patients with disorders of consciousness. Continued research and collaborative efforts between clinicians and researchers are necessary to optimize the outcomes of DBS therapy in this challenging patient population.

## 5 CONCLUSION

The medical community's concerted efforts in the field of DOC have led to significant advancements in neuroimaging and neuro‐modulation technologies, particularly in the application of DBS. However, challenges remain in standardizing DBS parameters and treatment protocols, and further research is needed to optimize its efficacy for individual patients.

Patient selection remains a critical factor in determining the success of DBS therapy for DOC. Accurate diagnosis and dynamic patient classification are essential to identify the most suitable candidates for intervention. Advanced paradigms based on PET, fMRI, and EEG hold promise for improving diagnostic accuracy and monitoring treatment outcomes, but their application as recommended clinical guidelines require further validation.

The selection of optimal DBS targets, such as the Centromedian‐parafascicular complex (CM‐pf) and the Central Thalamus (CT), has shown promising results in improving arousal levels and behavioral responsiveness. However, individualized approaches may be necessary to address the heterogeneity of patient populations and the underlying causes of DOC.

Stimuli parameter selection plays a crucial role in maximizing the beneficial effects of DBS while minimizing adverse outcomes. Adjusting stimulation parameters, such as frequency and pulse width, based on patient responses can lead to more effective therapy. Maintaining an optimal sleep‐wake cycle and consistent sleep patterns may also contribute to improved awake behavior in DOC patients undergoing DBS treatment.

Looking ahead, the future of DBS therapy for DOC holds great promise. Advances in neuroimaging techniques, including PET, fMRI, and EEG, will likely play a pivotal role in enhancing diagnostic accuracy and providing a more personalized approach to treatment. The ongoing development of innovative paradigms and biomarkers may further refine patient selection, leading to improved outcomes and better prognoses. As research in the field progresses, efforts to standardize DBS parameters and treatment protocols will likely yield valuable insights into optimizing the therapeutic potential of DBS for DOC patients. Comparative studies investigating the efficacy of different targets in specific patient subgroups based on etiology, duration of DOC, and severity will contribute to a more tailored and effective approach to DBS therapy.

The continued development of directional electrodes and advanced neuro‐modulation technologies will further enhance the precision and selectivity of DBS, potentially reducing adverse effects and increasing therapeutic efficiency. With continued dedication and exploration in this field, we can offer hope for improved outcomes and quality of life for those affected by DOC.

## AUTHOR CONTRIBUTIONS

Tianqing Cao: Conceptualization, Methodology, Validation, Formal analysis, Investigation, Writing—original draft, Writing—review & editing. Shenghong He: Formal analysis, Visualization. Xiaoke Chai: Methodology, Formal analysis, Data curation. Qiheng He: Methodology, Validation, Formal analysis, Writing—review & editing. Jianghong He: Writing—review & editing. Huiling Tan: Resources, Formal analysis, Writing—review & editing. Yi Yang: Writing—review & editing, Project administration, Funding acquisition. Jizong Zhao: Conceptualization, Software.

## CONFLICT OF INTEREST STATEMENT

The authors declare that they have no known competing financial interests or personal relationships that could have appeared to influence the work reported in this paper.

## Data Availability

The data supporting the results of this study can be obtained from authoritative databases (PubMed, Embase, Medline, and Web of Science), or you can contact the corresponding author to provide information.
